# HMGB1/2 can target DNA for illegitimate cleavage by the RAG1/2 complex

**DOI:** 10.1186/1471-2199-10-24

**Published:** 2009-03-24

**Authors:** Ming Zhang, Patrick C Swanson

**Affiliations:** 1 Department of Medical Microbiology and Immunology, Creighton University Medical Center, 2500 California Plaza, Omaha, NE, USA

## Abstract

**Background:**

V(D)J recombination is initiated in antigen receptor loci by the pairwise cleavage of recombination signal sequences (RSSs) by the RAG1 and RAG2 proteins via a nick-hairpin mechanism. The RSS contains highly conserved heptamer (consensus: 5'-CACAGTG) and nonamer (consensus: 5'-ACAAAAACC) motifs separated by either 12- or 23-base pairs of poorly conserved sequence. The high mobility group proteins HMGB1 and HMGB2 (HMGB1/2) are highly abundant architectural DNA binding proteins known to promote RAG-mediated synapsis and cleavage of consensus recombination signals *in vitro *by facilitating RSS binding and bending by the RAG1/2 complex. HMGB1/2 are known to recognize distorted DNA structures such as four-way junctions, and damaged or modified DNA. Whether HMGB1/2 can promote RAG-mediated DNA cleavage at sites lacking a canonical RSS by targeting or stabilizing structural distortions is unclear, but is important for understanding the etiology of chromosomal translocations involving antigen receptor genes and proto-oncogene sequences that do not contain an obvious RSS-like element.

**Results:**

Here we identify a novel DNA breakpoint site in the plasmid V(D)J recombination substrate pGG49 (bps6197) that is cleaved by the RAG proteins via a nick-hairpin mechanism. The bps6197 sequence lacks a recognizable heptamer at the breakpoint (5'-CCTGACG-3') but contains a nonamer-like element (5'-ACATTAACC-3') 30 base pairs from the cleavage site. We find that RAG-mediated bps6197 cleavage is promoted by HMGB1/2, requiring both HMG-box domains to be intact to facilitate RAG-mediated cleavage, and is stimulated by synapsis with a 12-RSS. A dyad-symmetric inverted repeat sequence lying 5' to the breakpoint is implicated as a target for HMGB1/2 activity.

**Conclusion:**

We have identified a novel DNA sequence, called bps6197, that supports standard V(D)J-type cleavage despite the absence of an apparent heptamer motif. Efficient RAG-mediated bps6197 cleavage requires the presence of HMGB1/2, is stimulated by synapsis with a 12-RSS partner, and is directed in part by an inverted repeat sequence adjacent to the DNA cleavage site. These results have important implications for understanding how the RAG proteins can introduce a DNA double-strand break at DNA sequences that do not contain an obvious heptamer-like motif.

## Background

Antigen receptor genes are assembled by a process known as V(D)J recombination from arrays of variable (V), diversity (D), and joining (J) gene segments that are flanked on one (V, J) or both (D) sides by a conserved recombination signal sequence (RSS) (for reviews, see [1-3]. The RSS contains a conserved heptamer element (consensus: 5'-CACAGTG-3') separated by either 12 or 23 base pairs (bps) from a conserved nonamer element (consensus: 5'-ACAAAAACC-3'). The RSS functions as the binding site for the RAG1 and RAG2 proteins, which together initiate V(D)J recombination by cleaving the RSS at the 5'-end of the heptamer through a two-step nick-hairpin mechanism. Normally, recombination only occurs between two gene segments that differ in the spacer length of the flanking RSS (one 12-RSS and one 23-RSS), a restriction termed the 12/23 rule. However, studies of recurrent breakpoint sequences identified in some types of lymphoid malignancies suggest that the RAG proteins may occasionally mediate illegitimate recombination by introducing a site-specific DNA break at a sequences resembling an RSS (a "cryptic" RSS or cRSS) [4-6], or catalyzing structure-specific cleavage at sites prone to adopting non-B form DNA conformations [7,8].

To be considered a plausible cRSS, it is generally thought that the putative cRSS must minimally contain at least the first three residues of the consensus heptamer (CAC) [9] due to the high degree of sequence conservation of these residues among *bona fide *RSSs [10] and based on functional studies of mutant RSS substrates which demonstrate that mutation of any of these residues essentially abolishes RAG-mediated cleavage [11-13] and V(D)J recombination [10]. The RAG proteins also exhibit structure-specific nicking of DNA, preferentially targeting transitions from single- to double-stranded DNA [7,11,12,14,15]. Nicks can lead to DNA double strand breaks if they are introduced on both DNA strands in close proximity. To study structure-specific nicking by the RAG proteins, most studies have artificially introduced transitions from single- to double-stranded DNA into DNA substrates by incorporating bp mismatches, bulges, flaps, or gaps. Only one example has been reported of an otherwise fully complementary double-stranded DNA adopting a non-B form DNA conformation that is targeted by the RAG complex for nicking [7]. Whether cellular factors can help stabilize alternative DNA conformations in otherwise complementary DNA which can then be targeted for RAG-mediated cleavage remains unclear.

In principle, high mobility group proteins that belong to the HMG-box family of architectural DNA binding and bending factors (e.g. HMGB1 or HMGB2) are plausible candidates for promoting mistargeted RAG activity because they are capable of binding structurally distorted DNA [16], such as four-way junctions [17] and damaged or modified DNA [18-20], and also can interact directly with the RAG proteins [21] and stimulate RAG-mediated RSS binding and cleavage activity *in vitro *[22,23] and V(D)J recombination in cell culture assays [21]. In support of this possibility, we have identified a novel DNA breakpoint site in the plasmid V(D)J recombination substrate pGG49 (bps6197) that is cleaved by the RAG proteins via a nick-hairpin mechanism in the presence of HMGB1/2. We find that RAG-mediated bps6197 cleavage yields a blunt end with the sequence 5'-CCTGACG-3' that is separated by 23-bps from a nonamer-like element (5'-ACATTAACC-3'). RAG cleavage activity at bps6197 is stimulated by synapsis with a 12-RSS, and requires both HMG-box domains of HMGB1 to be intact. Evidence is presented that HMGB1/2 targets a dyad-symmetric inverted repeat sequence lying 5' of the cleavage site. The implications of these findings are discussed.

## Results

### Identification of a novel RAG-mediated breakpoint sequence, bps6197, in pGG49 that lacks an obvious heptamer

**Figure 1:**
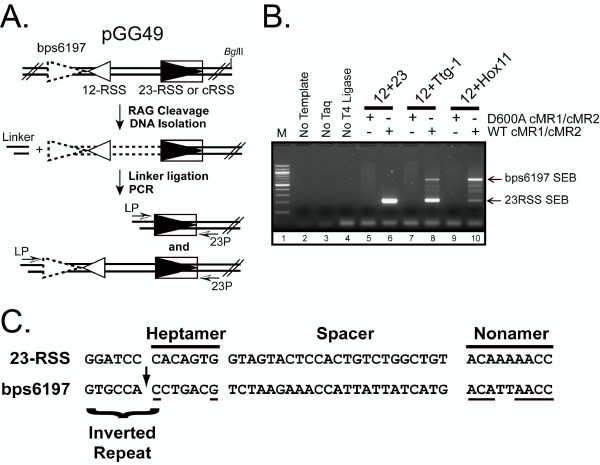
**Identification of bps6197 as a novel breakpoint in pGG49**. (A) Plasmid substrates cleaved by the RAG proteins *in vitro *are subjected to linker ligation followed by PCR using a linker primer (LP) and a primer proximal to the 23-RSS (23P) to detect signal end breaks (SEBs) at the 23-RSS (large filled triangle) or another adventitious site (open stippled triangle) upstream of the 12-RSS (small open triangle). (B) pGG49 (12/23) or its derivatives containing Ttg-1 or Hox11 sequences in place of the 23-RSS (12+Ttg-1 and 12+Hox11, respectively) were incubated with WT or D600A cMR1/cMR2 and HMGB1 in an *in vitro *cleavage reaction and SEBs were detected by ligation-mediated PCR (LM-PCR) as described in (A). The positions of LM-PCR products revealed on an agarose gel corresponding to SEBs at the 23-RSS or a novel sequence (bps6197) are indicated at right. (C) The bps6197sequence is shown aligned to the 23-RSS in pGG49. Nucleotides in bps6197 identical to the consensus 23-RSS heptamer and nonamer sequences are underlined. The site of cleavage is identified by an arrow. A dyad-symmetric inverted repeat at the heptamer-coding junction of bps6197 is also indicated.

In a previous study, Raghavan and Lieber characterized the V(D)J recombination potential of several putative cRSSs identified from lymphoid malignancies, including Ttg-1 and Hox11 [4]. To assay Ttg-1 and Hox11 recombination activity, the plasmid recombination substrate pGG49, which contains a consensus 12-RSS and a 23-RSS in a deletional configuration, was modified to replace the 23-RSS with Ttg-1 and Hox11 sequences (Fig. 1A). To follow up that study, we recently investigated *in vitro *RAG-mediated cleavage of these plasmid substrates, using ligation-mediated PCR (LM-PCR) to detect blunt signal end breaks (SEBs) at the 23-RSS or the cRSS [24] (Fig. 1A). Consistent with previous results, we find that SEBs are readily detected in plasmid substrates containing a consensus 23-RSS when incubated with wild-type but not catalytically inactive RAG proteins (WT and D600A cMR1/cMR2, respectively) in the presence of HMGB1, but the abundance of these breaks diminishes when the 23-RSS is replaced by the Ttg-1 sequence, and is reduced even further upon replacement with the Hox11 sequence (Fig. 1B). However, as cleavage at the cRSS diminishes, cleavage at an alternative site increases (Fig. 1B). Cloning and sequencing of the major LM-PCR product revealed a recurrent breakpoint at position 6197 (bps6197) of the plasmid substrate. Interestingly, this sequence lacks an obvious heptamer motif next to the breakpoint (5'-CCTGACG-3'), but contains a plausible nonamer-like element 30 nucleotides from the cleavage site (matching 7 of 9 consensus nucleotides), similar to a 23-RSS (Fig. 1C). We also noticed the presence of a seven bp dyad-symmetric inverted repeat sequence 5' of the breakpoint, overlapping the cleavage site by one bp at the 3' end. Because the sequence 3' to the breakpoint lacks consensus residues at positions equivalent to the second and third bps of the consensus heptamer, which previous studies have shown are necessary to support efficient RAG-mediated RSS cleavage [11-13] and V(D)J recombination [10], we wondered whether this site is cleaved by the RAG proteins through a standard nick-hairpin mechanism, and questioned whether the flanking inverted repeat element rather than the heptamer sequence might play a key role in guiding RAG-mediated cleavage at this site. To answer this question, we pursued further biochemical analysis of RAG-mediated bps6197 cleavage.

### RAG-mediated cleavage of bps6197 occurs through a nick-hairpin mechanism and is stimulated by synapsis with a 12-RSS

**Figure 2:**
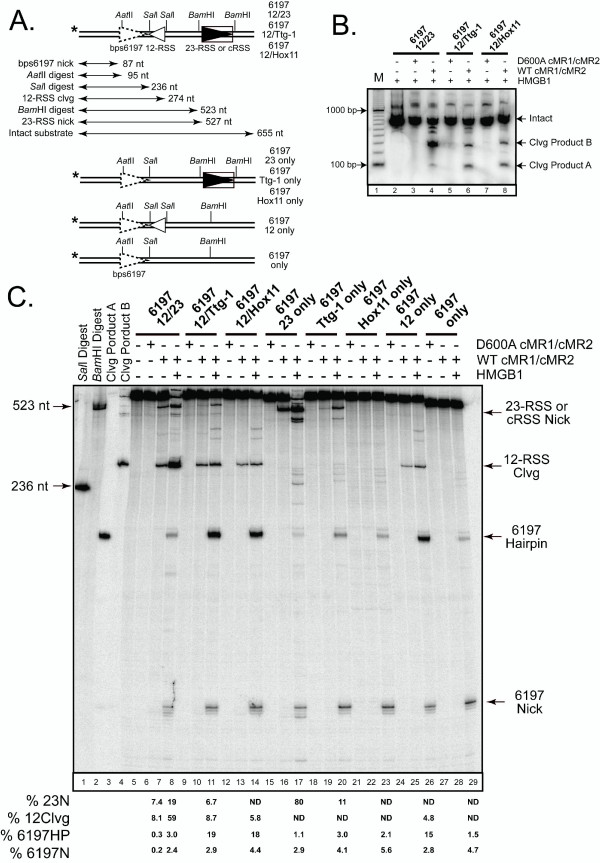
**Efficient RAG-mediated cleavage of bps6197 requires HMGB1 and is stimulated by synapsis with a 12-RSS partner**. (A) DNA fragments radiolabelled at the 5' end of the top strand (asterisk) were generated by PCR using pGG49 (bps6197/12/23) or its derivatives as templates (see diagrams; designations are indicated at right). The sizes of the intact bps6197/12/23 substrate and reaction products predicted from RAG-mediated cleavage or restriction enzyme digestion of this substrate are shown. (B) The indicated DNA fragments described in (A) were incubated in an *in vitro *cleavage reaction with HMGB1 and WT or D600A cMR1/cMR2 and purified reaction products were analyzed on a vertical agarose gel in parallel with a radiolabeled 100 bp ladder (M). Two major cleavage products (A and B, indicated at right) were isolated and used as markers in subsequent experiments. (C) The DNA fragments described in (A) were subjected to *in vitro *cleavage by WT or D600A cMR1/cMR2 in the absence or presence of HMGB1 as indicated. Reaction products were analyzed on a 40% formamide sequencing gel in parallel with markers derived from restriction enzyme digestion of bsp6197/12/23 or isolated after RAG-mediated cleavage as described in (B). Expected fragment sizes and compositions are indicated at left and right, respectively. The abundance of the major reaction products are quantified below the gel. Comparable results have been obtained in independent experiments.

To examine the bps6197 cleavage mechanism in more detail, we used PCR to prepare ~650 bp top strand radiolabeled DNA substrates from wild-type pGG49 and its derivatives containing Ttg-1 or Hox11 in place of the 23-RSS (6197/12/23, 6197/12/Ttg-1, and 6197/12/Hox11, respectively)(Fig. 2A). To determine whether these substrates are cleaved by the RAG proteins *in vitro*, we incubated the DNA fragments with WT or D600A cMR1/cMR2 and HMGB1 and separated the reaction products on a native agarose gel (Fig. 2B). As expected, RAG-mediated cleavage of the 6197/12/23 substrate yielded a major product of ~300 bp (cleavage product B) consistent with cleavage at the 12-RSS, and minor products of ~500 bp and ~100 bp (cleavage product A) expected from cleavage at the 23-RSS (or cRSS) and bps6197, respectively. Consistent with results obtained by LM-PCR, replacement of the 23-RSS with Ttg-1 or Hox11 sequences results in diminished *in vitro *cleavage at the cRSS, and a concomitant increase in bps6197 cleavage (Fig. 2B).

To follow up this experiment, we incubated the substrates with WT or D600A cMR1/cMR2 in the absence or presence of HMGB1 and analyzed the reaction products on a denaturing polyacrylamide gel in parallel with native gel-isolated cleavage products A and B as well as sizing markers derived from restriction endonuclease cleavage of the intact bps6197/12/23 substrate (see Fig. 2A). We find that cleavage product B migrates more slowly than a 236 nt fragment generated by *Sal*I digestion of the intact bps6197/12/23 substrate, and comigrates with the major reaction product generated by RAG-mediated cleavage (Fig. 2C, compare lane 4 to lanes 7 and 8). In contrast, cleavage product A migrates just moderately faster than the *Sal*I cleavage product, but much more slowly than a 95 nt product generated by *Aat*II digestion (not shown, but see Fig. 4 for example), and is only observed in the *in vitro *cleavage reactions containing WT cMR1/cMR2 and HMGB1 (Fig 2C, compare lane 3 to lanes 7 and 8). Moreover, the abundance of this product increases when the 23-RSS is replaced by Ttg-1 or Hox11 sequences, and this increase is correlated with reduced nicking at these cRSSs (Fig. 2C, compare lane 8 to lanes 11 and 14). Faster migrating reaction products are also detected that correspond to nicks introduced at positions 6195 and 6197 (see Additional File 1: Verification of nicking sites in PCR-generated substrates containing bps6197), with the latter product being more abundant than the former. The apparent doubling in the size of product A between native and denaturing gels and the detection of nicks at bps6197 provide compelling evidence that bps6197 supports RAG-mediated cleavage by a nick-hairpin mechanism.

The relationship between the level of nicking observed at the 23-RSS/cRSS and bps6197 cleavage activity suggested that the removing the 23-RSS itself may be sufficient to promote bps6197 cleavage. To test this possibility, and the role of the 12-RSS in supporting bps6197 cleavage activity, mutant substrates were prepared that lacked one or both RSSs (Fig. 2A) and then these substrates were subjected to *in vitro *cleavage by WT or D600A cMR1/cMR2 in the absence or presence of HMGB1 (Fig. 2C). We find that substrates lacking the 12-RSS support lower levels of bps6197 hairpin formation than their counterparts containing a 12-RSS (Fig. 2C, compare lanes 8, 11, and 14 to lanes 17, 20, and 23), but removal of the 23-RSS enhanced bps6197 cleavage relative to the 6197/12/23 substrate, with substrate cleavage levels similar to that observed with the 6197/12/Hox11 substrate (Fig 2C, compare lane 26 to lanes 8 and 14). In contrast, when both the 12- and 23-RSS were absent, bps6197 was cleaved poorly (Fig. 2C, lane 29). Taken together, these data strongly suggest that bps6197 cleavage is stimulated by synapsis with a 12-RSS partner. To determine whether the distance or orientation of the 12-RSS partner positioned *in cis *influences the efficiency of RAG-mediated bps6197 cleavage, we removed the proximal 12-RSS and replaced the distal 23-RSS with a 12-RSS in either orientation. We find that the level of RAG-mediated bps6197 cleavage of these substrates *in vitro *is quite similar to that observed with the 6197/12 substrate (see Additional File 2: RAG-mediated bps6197 cleavage is not affected by the distance or orientation of the 12-RSS partner), suggesting that RAG-mediated bps6197 cleavage is not affected by the distance or orientation of the 12-RSS partner.

### Analysis of RAG-mediated cleavage and binding of bps6197 using oligonucleotide substrates

**Figure 3:**
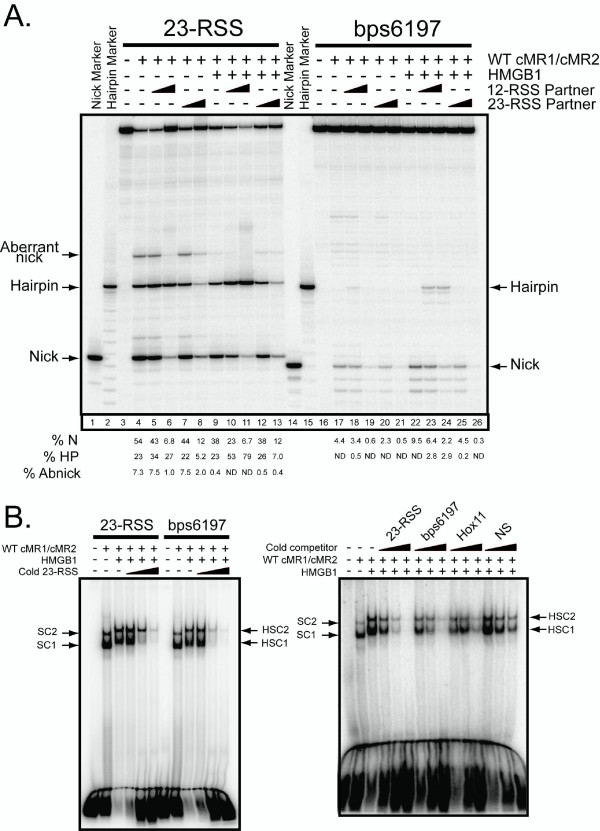
**Oligonucleotide substrates containing bps6197 are bound and cleaved by the RAG proteins *in vitro***. (A) Radiolabeled 23-RSS or bps6197 oligonucleotide substrates were incubated in an *in vitro *cleavage reaction containing WT cMR1/cMR2 with or without added HMGB1 and/or cold partner 12- or 23-RSS (0.1 or 1.0 pmol) as indicated. Reaction products were fractionated on a sequencing gel in parallel with radiolabeled markers corresponding to predicted nick and hairpin products. The percentage of appropriately sited nick (%N) and hairpin (%HP) products as well as aberrant nicks (%Abnicks) are quantified below the gel and are representative of independent experiments. (B) (Left panel) Radiolabeled 23-RSS or bps6197 oligonucleotide substrates were incubated with WT cMR1/cMR2 in binding reactions in the absence or presence of HMGB1 and increasing amounts (0.1, 1.0, or 10 pmol) of cold 23-RSS as a competitor. (Right panel) WT cMR1/cMR2 was incubated with a radiolabeled 23-RSS substrate with or without HMGB1 and increasing amounts (0.1, 1.0, or 1.0 pmol) of cold 23-RSS, bps6197, Hox11 or non-specific (NS) oligonucleotide substrates as a competitor. Protein-DNA complexes were separated by EMSA.

We were interested in determining whether stimulation of bps6197 cleavage by a 12-RSS paired *in cis *can be reconstituted *in trans *using oligonucleotide substrates. To test this possibility, we prepared 61 bp radiolabeled oligonucleotide substrates containing a consensus 23-RSS or the bps6197 sequence and incubated them with WT cMR1/cMR2 in reaction buffer containing Mg^2+ ^in the absence or presence of HMGB1 and/or cold 12-RSS or 23-RSS partner (Fig. 3A). As expected from our previous studies [24], the 23-RSS substrate is nicked robustly by the RAG proteins under these conditions, with some hairpin formation and aberrant nicking in the spacer also detected. Both appropriate and aberrant nicking is suppressed when only cold partner RSS is added or when HMGB1 is present (Fig. 3A, compare lane 4 to lanes 6 and 9). However, addition of both HMGB1 and cold 12-RSS partner stimulates RAG-mediated 23-RSS cleavage (hairpin formation), but cold 23-RSS diminishes the level of cleavage observed (Fig. 3A, compare lane 4 to lanes 11 and 13). In contrast to the 23-RSS, RAG-mediated bps6197 nicking is modestly stimulated (~2-fold) in the presence of HMGB1 (Fig. 3A, compare lanes 17 and 22). However, like the 23-RSS, addition of HMGB1 and cold 12-RSS, but not 23-RSS, promotes RAG-mediated bps6197 cleavage, although the amount of hairpin produced is ~20–25-fold lower than that observed for the consensus 23-RSS (Fig. 3A, compare lane 17 to lanes 24 and 26). These data support the conclusion that bps6197 functionally resembles a 23-RSS.

In principle, the lower cleavage activity observed with bps6197 relative to a consensus 23-RSS could reflect poorer RAG binding to this substrate. To test this possibility, we compared the RAG binding activity between the consensus 23-RSS and bps6197 substrates using an electrophoretic mobility shift assay (EMSA). In the first experiment, radiolabeled substrates were incubated with WT cMR1/cMR2 alone or with HMGB1 in the absence or presence of increasing amounts of cold 23-RSS as a specific competitor (Fig. 3B, left panel). In the absence of HMGB1, WT cMR1/cMR2 assembles two discrete protein-DNA complexes with a consensus 23-RSS. We have previously shown that these RAG-RSS complexes, called SC1 and SC2, both contain a RAG1 dimer and either one (SC1) or two (SC2) molecules of RAG2 [25]. Both complexes are supershifted by the addition of HMGB1, and the abundance of these complexes is diminished by further addition of increasing amounts of cold 23-RSS competitor. We find that the RAG proteins assemble the various protein-DNA complexes on the bps6197 substrate similarly to a consensus 23-RSS, with protein-DNA complex formation only slightly more susceptible to competition by a cold 23-RSS. To confirm these results, we assembled RAG complexes on a radiolabeled 23-RSS substrate in the presence of increasing concentrations of cold 23-RSS, bps6197, Hox11 or non-specific competitor DNA (Fig. 3B, right panel). We find that, as competitors, the substrates can be ordered from strongest to weakest as follows: 23-RSS>bps6197>Hox11>non-specific DNA, with differences between each being approximately 2–3 fold. Together, these data suggest that bps6197 is competent to support stable formation of RAG-DNA complexes *in vitro *to levels that are close (within ~2-fold) to a consensus 23-RSS.

### Site-specific bps6197 cleavage is directed by inverted repeat and nonamer-like sequences

**Figure 4:**
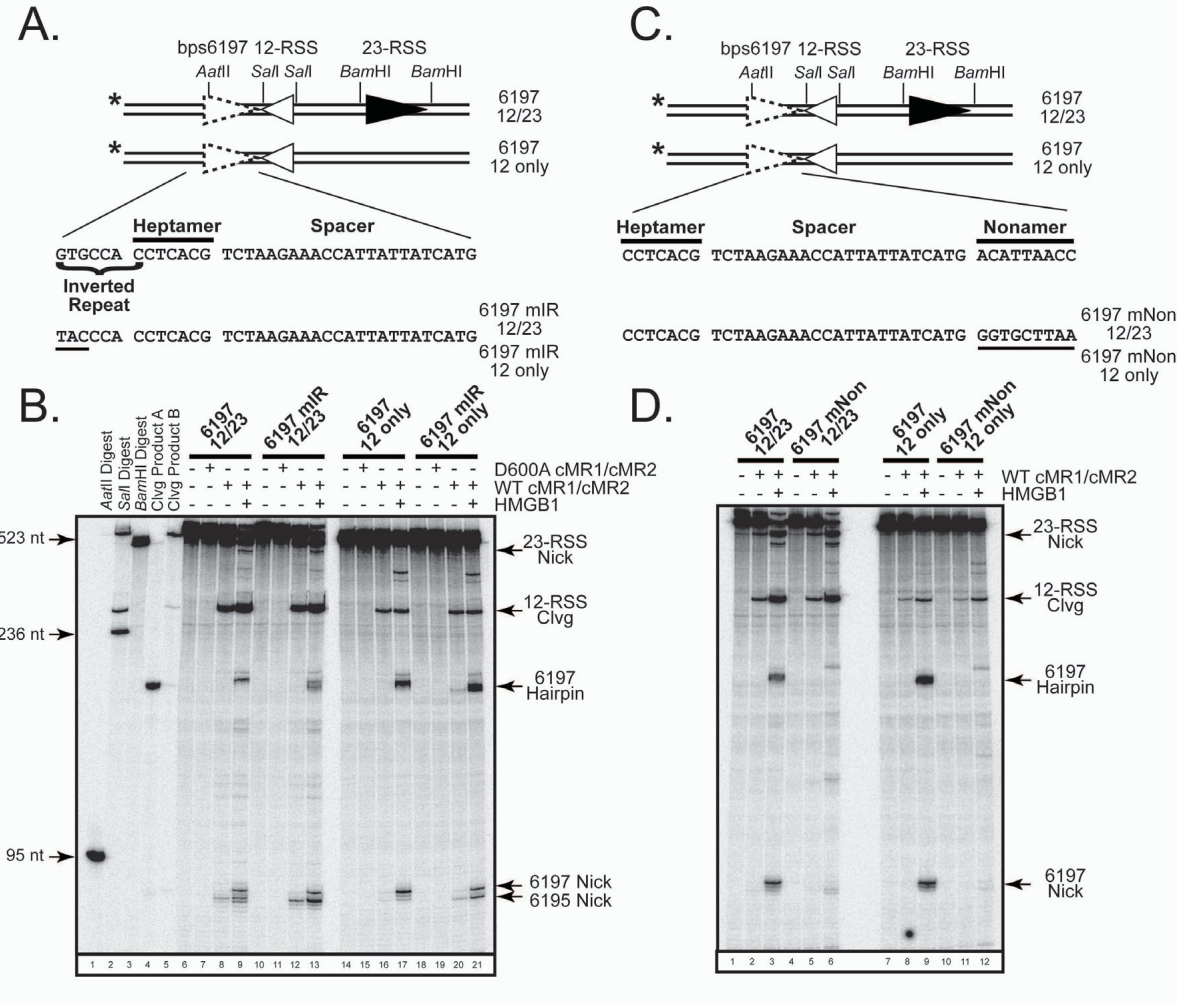
**RAG-mediated bps6197 cleavage is directed by the inverted repeat sequence and requires the nonamer-like sequence**. (A) DNA fragments radiolabeled at the 5'-end were generated by PCR using pGG49 containing or lacking a 23-RSS or their derivatives containing three bp substitutions in the inverted repeat distal to the cleavage site (underlined). (B) The DNA fragments in (A) were subjected to RAG-mediated cleavage as in Figure 2C. (C-D) Radiolabeled DNA fragments were prepared containing a wild-type or mutant (underlined) nonamer-like sequence in bps6197 as described in A and subjected to RAG-mediated cleavage as in (B). Independent experiments reveal similar results.

To directly test the sequence requirements for RAG-mediated bps6197 cleavage, we generated two mutant bps6197 substrates. In the first, the 5' end of the inverted repeat sequence was mutated (6197 mIR, see Fig. 4A) to determine whether this sequence influences where the RAG proteins cleave the substrate. If the location of bps6197 cleavage is directed solely by specific recognition of residues at positions equivalent to the heptamer, then one might expect mutating residues 4–6 nucleotides distal to the cleavage site in the coding flank would not appreciably alter where the RAG proteins cleave the substrate. Interestingly, however, we find that mutation of these residues shifts the preferred cleavage site by two nucleotides, so that a CAC trinucleotide sequence (6195), which is also found in a consensus heptamer, is preferentially nicked compared to the CCT trinucleotide sequence (6197) targeted in the wild-type bps6197 substrate (Fig. 4B, compare lane 9 to lane 13). This observation also holds true if the substrates lack the 23-RSS (Fig 4B, compare lane 17 to lane 21). These data suggest that the coding flank sequence in bps6197 plays a larger role in directing the site of cleavage than in a consensus RSS, where residues in the heptamer play a predominant role in guiding the position of RAG-mediated cleavage [11-13]. To determine whether the nonamer-like sequence identified in bps6197 is required to support bps6197 cleavage, we mutated the putative nonamer motif (6197 mNon, see Fig. 4C) and analyzed reaction products generated after RAG-mediated cleavage of this substrate. We find that mutating this element largely abolishes nicking and hairpin formation at bps6197, regardless of whether the consensus 23-RSS paired *in cis *is present or absent in these substrates (Fig. 4D, compare lanes 3 and 9 to lanes 6 and 12).

**Figure 5:**
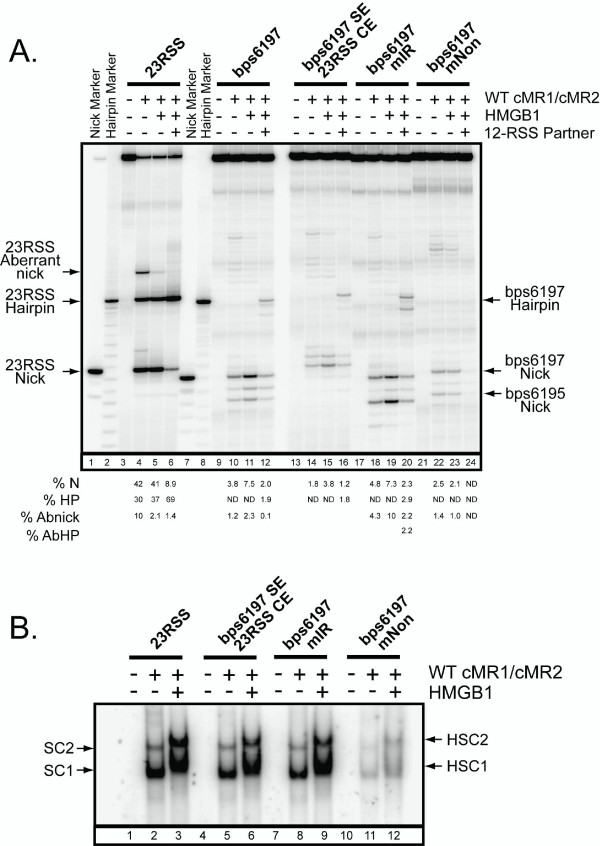
**The effect of bps6197 mutations is reconstituted using oligonucleotide substrates**. (A) A radiolabeled 23-RSS substrate, a wild-type bps6197 oligonucleotide substrate, or bps6197 substrates bearing a 23-RSS coding flank substitution (bps6197 SE/23RSS CE) or mutations in the inverted repeat (bps6197 mIR) or nonamer (bps6197 mNon) sequences were incubated in an *in vitro *cleavage reaction containing WT cMR1/cMR2 with or without added HMGB1 and cold partner 12-RSS (1.0 pmol) as indicated. Reaction products were analyzed as in Figure 3A. (B) WT cMR1/cMR2 was incubated with the radiolabeled substrates in (A) in binding reactions lacking or containing HMGB1. Protein-DNA complex formation was analyzed by EMSA as in Figure 3B.

Having established a functional role for the inverted repeat and nonamer-like sequences in supporting RAG-mediated bps6197 cleavage on PCR-generated substrates, we wished to determine whether the effects of substrate mutations on RAG-mediated cleavage of long DNA fragments can be recapitulated using short oligonucleotide substrates, and to evaluate the degree to which substrate mutations impaired RAG-DNA complex formation. In general, we find that mutations in the inverted repeat and nonamer-like sequences show comparable effects on the reaction product profile after RAG-mediated cleavage as their counterpart long DNA fragments, except that there is less dependence on the presence of HMGB1 for the introduction of nicks in the bps6197 sequence (Fig. 5A). Specifically, mutations in the inverted repeat were found to skew the pattern of reactivity in favour of cleavage two nucleotides into the coding flank, with hairpins derived from both nicks being clearly evident in the presence of HMGB1 and cold 12-RSS partner (Fig. 5A, compare lanes 10–12 to lanes 18–20). In contrast, mutations in the nonamer-like sequence greatly reduce substrate cleavage activity under the same conditions (Fig. 5A, compare lanes 10–12 to 22–24). To examine whether the flanking sequence itself is required to direct RAG-mediated cleavage, we also tested a mutant oligonucleotide substrate in which the sequence flanking bps6197 is replaced by the coding flank of the consensus 23-RSS. Interestingly, this sequence shows no evidence of RAG-mediated nicking in the coding flank, but supports levels of nicking and hairpin formation at the predicted heptamer-coding junction that are modestly lower than the wild-type bps6197 substrate (Fig. 5A, compare lanes 10–12 to lanes 14–16). The differential cleavage of substrates bearing mutations in the coding flank are not attributed to differences in how these substrates are bound by the RAG proteins, because the abundance and distribution of RAG-DNA complexes assembled on these substrates is quite similar to those formed using a consensus 23-RSS substrate, as assessed by EMSA (Fig. 5B). However, mutation of the nonamer-like sequence clearly impairs RAG-DNA complex formation. Taken together, these data suggest that the coding sequence of bps6197 plays an important role in directing the site of RAG-mediated cleavage and that the nonamer-like element functions to stabilize RAG binding to this DNA sequence.

### Determinants of HMGB1 required to promote RAG-mediated bps6197 cleavage

The dependence of RAG-mediated bps6197 cleavage in long DNA fragments on HMGB1 led us to question what determinants of HMGB1 were required to support this activity. HMGB1 and its vertebrate homologue HMGB2, contain tandem HMGB-box domains (called A and B) and an acidic C-terminal tail separated from the HMG-boxes by a linker sequence rich in basic residues [16]. Both HMG-box domains share a globally similar architecture comprised of three alpha helices that adopt an L-shaped structure. Both domains possess DNA binding activity, primarily interacting with the minor groove and mediating DNA bending in part by intercalating hydrophobic residues between DNA bps. Despite this similarity, the two domains exhibit distinct DNA binding preferences: box A selectively binds distorted DNA structures, whereas box B shows less preference for such structures but, unlike box A, can itself induce a severe bend in linear DNA. The functional activity of the HMG-box domains is further modulated in various ways by the C-terminal basic and acidic residues.

**Figure 6:**
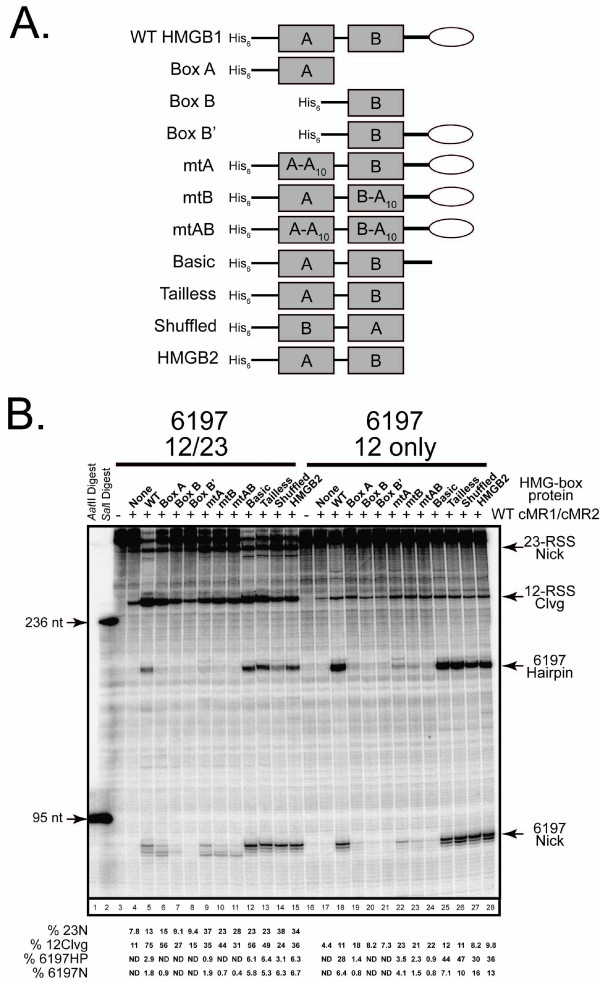
**Single HMG-box domains and forms of HMGB1 bearing mutations in regions required for its DNA binding and bending activity fail to efficiently stimulate RAG-mediated bps6197 cleavage**. (A) Diagrams of full-length, truncated, and mutant forms of recombinant HMGB1 and HMGB2 used in this study. The amino-terminal polyhistidine tag (His_6_), the HMG box domains (rectangles), the basic linker (heavy line) and the acidic tail (oval) are indicated schematically. Truncation mutants are shown comprising either a single HMG-box A domain (Box A) or a single HMG-box B domain lacking or containing the basic linker and acidic tail (Box B and B', respectively). Other truncation mutants containing both HMG-box domains lacking the acidic tail only (Basic) or both the basic linker and acidic tail (Tailless) are also shown. A form of Tailless HMGB1 in which the order of the HMG-box domains is reversed was also prepared (Shuffled). Mutant forms of full-length HMGB1 contain ten consecutive alanine substitutions (A_10_) in box A (mtA, residues 18–27), box B (mtB, residues 102–111), or both (mtAB). (B) The indicated radiolabeled DNA fragments were incubated with WT cMR1/cMR2 in an *in vitro *cleavage reaction lacking or containing the various forms of HMGB1 or HMGB2 shown in (A). Based on previous studies [26], different amounts of each form of HMGB1 or HMGB2 was added to the cleavage reaction as follows: 600 ng (Box B'), 300 ng (WT, mtA, mtB, and mtAB), 200 ng (Box A and Box B), or 100 ng (Basic, Tailless, Shuffled, and HMGB2). Similar results were obtained in independent experiments.

Given the distinct functional properties of the different regions of HMGB1, we sought to examine their roles in stimulating RAG-mediated bps6197 cleavage. In a previous study to identify determinants of HMGB1 required to stimulate RAG-mediated cleavage of an oligonucleotide substrate containing a consensus 23-RSS, we prepared a large panel of truncated wild-type and mutant forms of HMGB1 [26](Fig. 6A). We evaluated this panel of HMGB1 proteins, as well as a truncated form of wild-type HMGB2, for their ability to promote RAG-mediated bps6197 cleavage in long DNA fragments amplified from pGG49 or its derivative lacking the 23-RSS (Fig. 6B). We find that, compared to full-length wild-type HMGB1, individual HMG-box domains and forms of HMGB1 containing mutations in one or both HMG-boxes that target residues involved in mediating key protein-DNA interactions show severe defects in stimulating RAG-mediated bps6197 cleavage, regardless of whether or not a partner consensus 23-RSS is present in the substrate *in cis*. Interestingly, except for Box B', these proteins remain capable of stimulating 12-RSS cleavage in these substrates relative to samples containing cMR1/cMR2 alone. This outcome is consistent with our recent data showing that despite failing to stimulate RAG-mediated cleavage on an isolated 23-RSS oligonucleotide substrate in Mg^2+^, a single HMG-box domain can promote 23-RSS cleavage when a partner 12-RSS is present [27]. In this study, either the 23-RSS or bps6197 may serve *in cis *as the partner to the 12-RSS in the long DNA substrate. For the latter case, these data argue that bps6197 need not necessarily undergo nicking to stimulate 12-RSS cleavage. Consistent with previous studies [26], forms of HMGB1 lacking the acidic tail promote greater nicking and comparable to slightly higher levels of RAG-mediated bps6197 cleavage than full-length HMGB1. Interestingly, however, rearranging the order of HMG-boxes (AB vs BA) does not diminish the level of RAG-mediated bps6197 nicking, but does slightly impair hairpin formation at this site. This effect is also observed at the 12-RSS, suggesting that the two domains do not have entirely redundant functions in facilitating hairpin formation at RSSs positioned *in cis*. Finally, the ability of tandem HMG-box domains to stimulate RAG-mediated bps6197 cleavage is not unique to HMGB1, as a tailless form of HMGB2 also promotes cleavage at levels comparable to HMGB1.

### Bps6197 supports enhanced binding by HMGB1 compared to a 23-RSS

**Figure 7:**
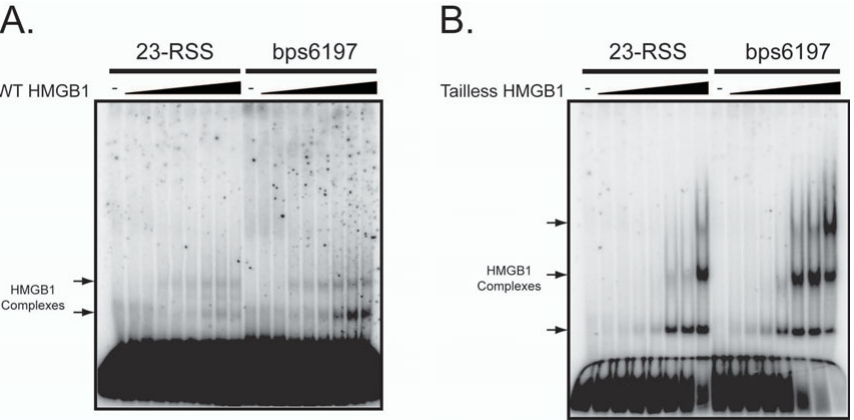
**HMGB1 exhibits preferential binding to bps6197 relative to a 23-RSS**. (A-B). Radiolabeled oligonucleotide substrates containing a consensus 23-RSS or bps6197 were incubated under binding conditions with increasing amounts of (A) full-length HMGB1 (0–320 ng in 40 ng increments; approximate protein:DNA ratio of 0–642:1) or (B) tailless HMGB1 (0–140 ng in 20 ng increments; approximate protein:DNA ratio of 0–328:1). Protein-DNA complex formation was analyzed by EMSA as in Figure 3B.

The strong dependence of RAG-mediated bps6197 cleavage on HMGB1 raises the possibility that HMGB1 selectively targets this sequence, and facilitates formation of a stable protein-DNA complex with the RAG proteins. If so, HMGB1 might be expected to bind bps6197 better than a consensus 23-RSS. To test this possibility, we incubated oligonucleotide substrates containing a consensus 23-RSS or bsp6197 with increasing amounts of wild-type full-length or tailless forms of HMGB1 and analyzed protein-DNA complex formation by EMSA (Fig. 7). We find that both forms of HMGB1 require about 1.5-fold less protein to form comparable levels of protein-DNA complexes on bps6197 compared to the 23-RSS, suggesting that HMGB1 preferentially binds bps6197 relative to the 23-RSS.

### Cleavage and recombination activity of bps6197 in cells

**Figure 8:**
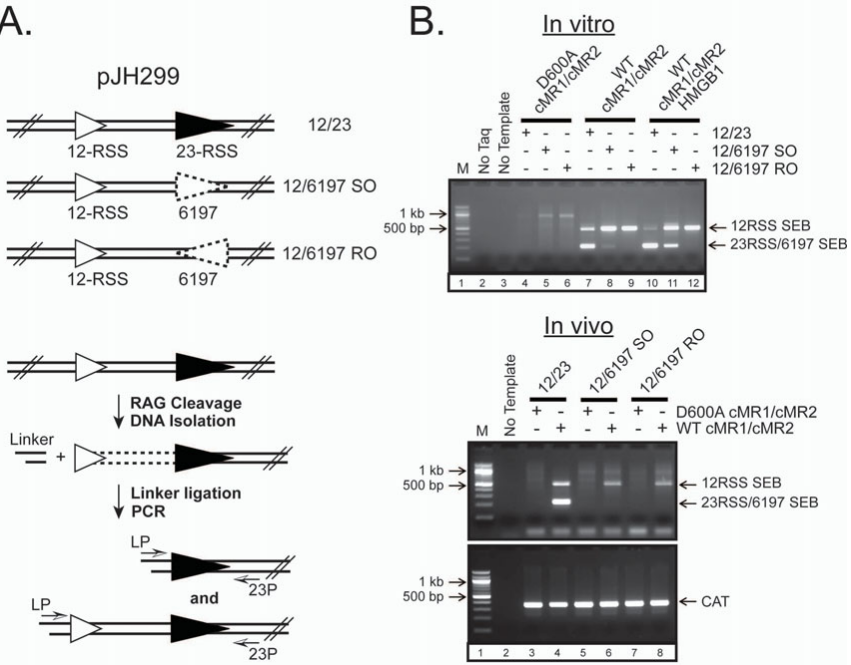
**RAG-mediated bps6197 cleavage in cells is not detectable**. (A) Diagrams showing the plasmid V(D)J recombination substrate pJH299 (12/23) or its derivatives in which the 23-RSS is replaced by bps6197 in the same orientation (12/6197SO) or reverse orientation (12/6197RO). SEBs produced by RAG-mediated cleavage were detected by LM-PCR as described in Figure 1. (B) (top) The plasmid substrates in (A), linearized with *Aat*II, were incubated with D600A or WT cMR1/cMR2 in an *in vitro *cleavage reaction lacking or containing HMGB1, and RSS or bps6197 SEBs were detected by LM-PCR as in Figure 1B. (Bottom) Plasmid V(D)J recombination substrates in (A) were cotransfected with WT or D600A cMR1 and WT cMR2 expression constructs into 293 cells. Plasmid DNA recovered 72 h post-transfection was analyzed for the presence of RSS or bps6197 SEBs by LM-PCR using the strategy in (A). To verify the presence of template DNA, a fragment of the chloramphenicol acetyl transferase (CAT) gene in the pJH299 backbone was amplified by PCR as a control.

Since *in vitro *studies clearly establish that bps6197 is a target for RAG-mediated cleavage in the presence of HMGB1, we wondered whether this site supports V(D)J recombination in cells. To examine this possibility, we generated plasmid V(D)J recombination substrates, derived from pJH299, containing a 12-RSS and either a consensus 23-RSS in the same orientation as the 12-RSS (12/23) or bps6197 in the same or reverse orientation relative to the 12-RSS (12/6197SO and 12/6197RO) (Fig. 8A). The pJH299 backbone was used instead of pGG49 in this case due to concerns about removing the bps6197 sequence from its native context in the lac promoter 5' of the 12-RSS. We first wished to test whether bps6197 is a target for RAG-mediated cleavage in the context of pJH299 *in vitro*. We find that, consistent with results in Figure 1, bps6197 SEBs are detected by LM-PCR in 12/6197SO plasmid DNA recovered after *in vitro *cleavage by WT, but not D600A cMR1/cMR2; SEBs at this site increase when HMGB1 is also present in the cleavage reaction (Fig. 8B, top panel, compare lanes 8 and 11). SEBs are not detected when bps6197 is in the opposite orientation (12/6197RO), as expected if RAG-mediated cleavage yields a covalently sealed coding end proximal to the 23P primer that cannot be ligated to linker DNA (Fig. 8B top panel; e.g., compare lanes 11 and 12). Cleavage at the 12-RSS is also detected in these assays, and increases when the 23-RSS is replaced by bps6197.

Having established that the RAG proteins can cleave bps6197 when embedded in the pJH299 backbone, we next cotransfected these substrates with WT or D600A cMR1 and WT cMR2 expression constructs in 293 cells and analyzed plasmid DNA recovered 72 h after transfection for bps6197 and/or RSS SEBs. We find that SEBs at both RSSs are readily detected in the recovered 12/23 substrate cotransfected with WT cMR1/cMR2, but not D600A cMR1/cMR2 (Fig. 8B, bottom panel). However, in contrast to *in vitro *cleavage assays, bsp6197 SEBs are not observed in the 12/6197SO plasmid DNA recovered from cell culture, despite detecting cleavage at the 12-RSS positioned *in cis *(which occurs at lower levels than in the 12/23 substrate). We considered the possibility that HMGB1 expression may be insufficient in 293 cells to promote RAG-mediated bps6197 cleavage. However, HMGB1 is readily detected in 293 cell lysates and repeating these assays in 293 cell lines overexpressing HMGB1 did not change the outcome of the experiment (data not shown). Therefore, we conclude that bps6197 is not cleaved by the RAG proteins in cells to levels that are within the detection limit of our LM-PCR assay. We consider possible explanations for this result below.

## Discussion

Here we describe the identification of a novel breakpoint sequence in pGG49 called bsp6197 that is targeted for RAG-mediated cleavage *in vitro *in the presence of HMGB1. Because the sequence lacks an obvious heptamer motif and possesses an inverted repeat in the sequence flanking the breakpoint, we were curious about the mechanism underlying the cleavage reaction and considered the possibility that RAG-mediated cleavage is directed by the inverted repeat. We show here that bps6197 supports RAG-mediated cleavage via a nick-hairpin mechanism. Efficient RAG-mediated bps6197 cleavage depends on the presence of HMGB1 and synapsis with a partner 12-RSS, and is guided in part by the inverted repeat sequence. To our knowledge, this is the first example of a flanking inverted repeat sequence influencing where the RAG proteins initiate DNA cleavage.

Given the dependence of RAG-mediated bps6197 cleavage on synapsis with a 12-RSS, we might have expected to detect LM-PCR products resulting from linker ligation to SEBs at both bps6197 and the 12-RSS, using the linker primer as both a forward and reverse primer. The predicted size of this LM-PCR product is 236 bp, which is 24 bp longer than the LM-PCR product that identifies the 23-RSS SEB using the linker primer and the primer downstream of the 23-RSS (23P, see Fig. 1A). Close inspection of Figure 1B does show the presence of low levels of LM-PCR products running slightly larger than those detecting the 23RSS SEB that are consistent with the introduction of SEBs at both the 12-RSS and bps6197. Their abundance may be less than one might expect for several possible reasons, including a lower probability of linker ligation at both SEBs, amplification from substrates containing bps6197 SEBs associated with a nicked 12-RSS (which would not be detected on the denaturing gels in Fig. 2C), and/or competition with PCR products amplified using the linker primer and 23P primer.

In our previous study, we found that RAG-mediated nicking and cleavage of oligonucleotide substrates containing cRSSs identified from lymphoid malignancies were either unchanged or slightly reduced in the presence of HMGB1 for most cRSSs tested (including LMO2, TAL1, Hox11, SIL, SCL), except Ttg-1 [24]. In the latter case, nicks at the predicted heptamer (which contains a fully consensus sequence) was not enhanced by addition of HMGB1, but nicks at other locations in the Ttg-1 sequence increased when HMGB1 was added to the cleavage reaction. In contrast, RAG-mediated nicking of a bps6197 oligonucleotide substrate increases with addition of HMGB1, and efficient nicking and hairpin formation at this site in long DNA is HMGB1-dependent.

One possible reason why RAG-mediated bps6197 cleavage shows a more stringent requirement for HMGB1 than other cRSSs examined previously is that HMGB1 may target and/or stabilize a structural distortion in the flanking inverted repeat sequence. This possibility is made plausible by previous studies showing that HMGB1 binds four-way junctions [17] and is further supported by evidence presented here that HMGB1 binds bps6197 better than a consensus 23-RSS. However, attempts to detect a stable pre-existing or protein-induced four-way junction in the inverted repeat of bps6197 using DNA footprinting experiments failed to yield compelling evidence for the existence of such a structure (data not shown), but we point out that this may be difficult to detect due to its small size, or if the structure is transient, limited in abundance, or masked in the bound complex. Similarly, although the lack of detectable bps6197 cleavage in cell culture experiments could be interpreted to mean that the RAG proteins fail to recognize this sequence *in vivo*, it is possible that factors bound to the plasmid substrate in cells render the site inaccessible to the RAG and/or HMGB1/2 proteins or unable to adopt a conformation that is targeted by HMGB1/2.

Although authentic RSSs in antigen receptor loci are the normal targets of the RAG proteins during V(D)J recombination, illegitimate RAG activity has been implicated in the etiology of chromosomal abnormalities recurrent in certain forms of leukemia and lymphoma [9]. A subset of these events have been attributed to the RAG proteins mistargeting a sequence resembling an RSS, and mediating a standard V(D)J-type rearrangement between an authentic RSS and a cRSS. In cases where there is clear evidence for this type of rearrangement, the cRSS contains at least the first three residues of the consensus heptamer (5'-CAC). A second subset of events has been suggested to occur through the illegitimate repair of a mechanistically undefined DNA double strand break with DNA ends produced by RAG-mediated cleavage at a pair of authentic RSSs. The source of the undefined DNA break in the second type of recombination event is unclear as these sites generally do not have a recognizable cRSS with a plausible heptamer motif. The data presented here raise the possibility that such sites, despite lacking an obvious heptamer, could nevertheless be subjected to illegitimate RAG-mediated cleavage if they contain a nonamer-like sequence that could anchor the RAG complex in proximity to the breakpoint. By recognizing or stabilizing a structural distortion at the breakpoint, HMGB1/2 may then promote illegitimate DNA cleavage by targeting the anchored RAG complex to the breakpoint in lieu of a consensus heptamer. It is likely that stable HMGB1/2 association with the breakpoint depends on concomitant interactions with the RAG proteins themselves, most likely RAG1 [21], as HMGB1 alone, despite displaying some sequence preference for bps6197 over the 23-RSS, nevertheless binds this site relatively weakly (Fig. 7). This targeting mechanism need not be unique to HMGB1/2, as it could also be facilitated directly or indirectly by transcription factors that bind in or near the breakpoint sequence and interact with the RAG proteins as an illegitimate form of an otherwise normal process [28,29].

## Conclusion

We have uncovered an example of illegitimate DNA cleavage by the RAG proteins at a DNA sequence called bps6197 that lacks an apparent heptamer motif. We demonstrate that bps6197 functionally resembles a 23-RSS and that RAG-mediated cleavage at this site: (i) occurs through a nick-hairpin mechanism; (ii) depends on the presence of HMGB1/2; (iii) is stimulated by synapsis with a 12-RSS; and (iv) is partly directed by an inverted repeat sequence 5' of the breakpoint. We provide evidence that HMGB1 alone binds bps6197 better than a 23-RSS, and that efficient RAG-mediated cleavage of this site requires both HMG-box domains to be intact. Taken together, these data suggest that HMGB1/2 can target illegitimate RAG-mediated cleavage at sequences lacking an evident heptamer. These results raise the possibility that breakpoint sequences identified in lymphoid malignancies that were previously thought unlikely to undergo RAG-mediated cleavage due to the absence of a cRSS with a heptamer-like sequence may nevertheless support cleavage by the RAG proteins in the presence of cofactors like HMGB1/2.

## Methods

### RAG and HMGB1 protein purification

Truncated and full-length forms of RAG1 (residues 384–1040 and 1–1040, respectively) and RAG2 (residues 1–387 and 1–517, respectively) containing an amino-terminal maltose binding protein fusion partner (cMR1, FLMR1, cMR2, and FLMR2, respectively) were coexpressed in 293 cells and purified by amylose affinity chromatography according to published procedures [30]. Full-length, truncated, or mutant forms of amino-terminal polyhistidine-tagged HMGB1 were expressed in *E. coli *and purified as described elsewhere [26].

### DNA substrates

Oligonucleotide substrates containing a 12-RSS or 23-RSS cRSS labelled at the 5' end of the top strand were prepared as described elsewhere [24,30]. A substrate containing the bps6197 sequence was similarly prepared from the oligonucleotide 5'-TCCCCGAAAAGTGCCACCTGACGTCTAAGAAACC ATTATTATCATGACATTAACCTATAAA-3' and its complement (positions equivalent to the 23-RSS heptamer and nonamer motifs are underlined). Derivatives of this substrate containing mutations at positions indicated in the text were also prepared. Unlabeled DNA substrates containing consensus 12-RSS, 23-RSS, Hox11 or non-specific DNA sequences (DAR81/82) were prepared from oligonucleotides described previously [11,24].

The plasmid V(D)J recombination substrates pGG49, its derivatives containing Ttg-1 or Hox11 cRSS sequences, and pJH299 have been described elsewhere [4,10]. Derivatives of pGG49 lacking the 12-RSS or 23-RSS were generated by *Sal*I or *BamH*I digestion, respectively. Variants of pGG49 and pJH299 containing the wild-type or mutant bps6197 sequences discussed in the text were generated according to procedures described in Additional File 3: Supplementary Materials and Methods. Long DNA fragments (~650 bp) radiolabeled at the 5' end of the top strand containing the RSS, cRSS, and/or wild-type or mutant bps6197 sequences present in pGG49 and its derivatives were generated by PCR using radiolabeled primer 6000F (5'-TATTGTCCTCATGAGCGGATAC-3') and unlabeled primer 6624R (5'-GAACGGTGGTATATCCAGTG-3'). PCR samples (100 μl final volume) containing plasmid template DNA (70 ng) and primers (20 pmol each) were subjected to initial denaturation at 95°C for 4 min, followed by 30 cycles of amplification (95°C for 45 sec, 56°C for 30 sec, and 72°C for 60 sec) and a final extension (72°C for 4 min). PCR products were isolated using a QIAquick PCR purification kit (Qiagen), eluted in 50 μl buffer EB (10 mM Tris, pH 8.0), fractionated on a vertical agarose gel (1.4%) at 150V for 1.5 h, and gel-isolated DNA purified using a QIAquick Gel Extraction Kit (Qiagen).

### *In vitro *RAG cleavage and binding assays

RAG-mediated cleavage of oligonucleotide substrates, PCR-generated long DNA fragments, or plasmid DNA was analyzed using an *in vitro *cleavage assay described previously [24]. Unless otherwise noted, basic *in vitro *cleavage reactions (10 μL) contained cMR1/cMR2 (~100 ng) and substrate DNA (~0.02 pmol radiolabeled oligonucleotide, ~250 ng long DNA substrate or100 ng unlabeled *Bgl*II-digested plasmid DNA) in reaction buffer containing Mg^2+^. Reactions were further supplemented with various forms of HMGB1 without or with additional unlabeled 12-RSS or 23-RSS partner (1 pmol) as indicated. Cleavage reactions were incubated at 37°C for 1 h. For some reactions containing long radiolabeled DNA fragments (e.g. Fig. 2B), DNA was purified using a QIAquick PCR purification kit, and reaction products separated under native conditions on a vertical 1% agarose gel. For other samples containing radiolabeled DNA, reactions were terminated by adding 2 volumes of sample loading solution (95% formamide, 10 mM EDTA) and reaction products fractionated under denaturing conditions on a 15% polyacrylamide sequencing gel lacking formamide (oligonucleotides) or an 8% polyacrylamide gel containing 40% formamide (long DNA fragments). Reaction products were analyzed using a Storm 860 phosphorimager running the ImageQuaNT software. For reactions containing unlabeled plasmid DNA (e.g. Fig. 1B), samples were diluted by adding 20 μL TE (10 mM Tris [pH 8.0], 1 mM EDTA], and heat inactivated for 10 min at 65°C. From 4 μL of this sample, SEBs generated at the 23-RSS or its replacement were detected using LM-PCR as previously described [24,31]. The formation of RAG and HMGB1 protein-DNA complexes on oligonucleotide substrates was analyzed using an EMSA as previously described [30].

## Abbreviations

RAG1: Recombination Activating Gene-1; RAG2: Recombination Activating Gene-2; HMGB1: High Mobility Group Box 1; HMGB2: High Mobility Group Box 2; bps6197: breakpoint sequence 6197; EMSA: electrophoretic mobility shift assay; LM-PCR: ligation-mediated PCR; bp: base-pair.

## Authors' contributions

PCS conceived the study and MZ performed all experiments. PCS drafted the manuscript, which was edited into final form with input from MZ. All authors have read and approved the final manuscript.

## Supplementary Material

Additional file 1 **Verification of nicking sites in PCR-generated substrates containing bps6197. **(A) Diagrams of PCR-generated substrates subjected to RAG-mediated cleavage *in vitro *and primer sets used to identify nicking sites in bps6197 with a wild-type or mutant inverted repeat sequence. (B) Sequencing gel showing reaction products from RAG-mediated cleavage of PCR-generated substrates. Click here for file

Additional file 2 **RAG-mediated bps6197 cleavage is not affected by the distance or orientation of the 12-RSS partner. **(A) Diagrams of PCR-generated substrates containing bsp6197 and a 12-RSS *in cis *positioned proximally or distally in the same or reverse orientation. (B) Sequencing gel showing reaction products from RAG-mediated cleavage of PCR-generated substrates. Click here for file

Additional file 3 **Supplementary Materials and Methods. **Construction of variants of pGG49 and pJH299 plasmid V(D)J recombination substrates described in the text. Click here for file
