# Supplementary material for: HMGB1/2 can target DNA for illegitimate cleavage by the RAG1/2 complex
Source: BMC Mol Biol. 2009 Mar 24;10:24. doi: 10.1186/1471-2199-10-24 (PMC2666730; doi:10.1186/1471-2199-10-24)
Supplement: Additional file 1 — Verification of nicking sites in PCR-generated substrates containing bps6197. (A) Diagrams of PCR-generated substrates subjected to RAG-mediated cleavage in vitro and primer sets used to identify nicking sites in bps6197 with a wild-type or mutant inverted repeat sequence. (B) Sequencing gel showing reaction products from RAG-mediated cleavage of PCR-generated substrates. [file 1471-2199-10-24-S1.pdf]

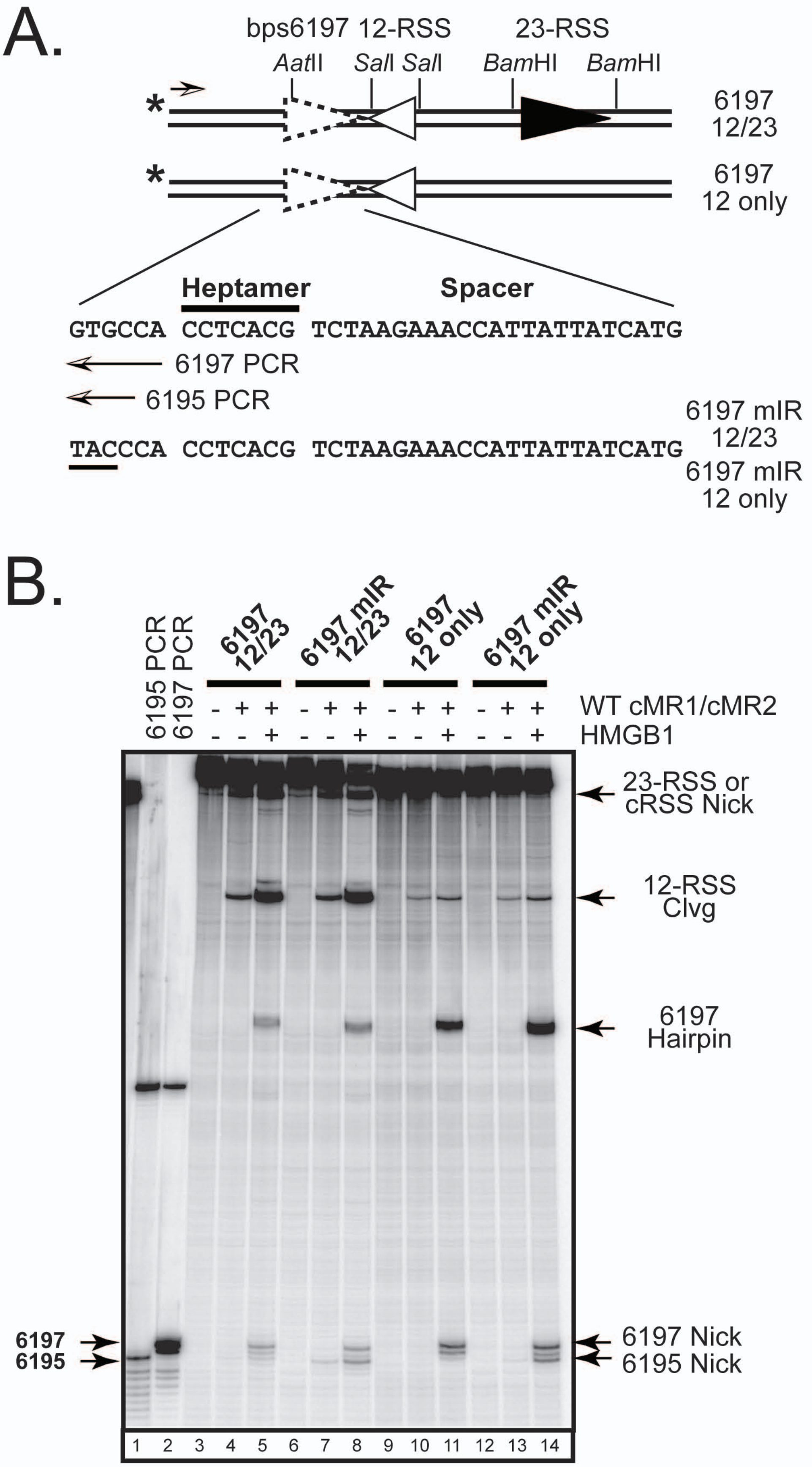

Additional Data File 1. Verification of nicking sites in PCR-generated substrates containing bps6197. (A) DNA fragments radiolabelled at the 5′ end of the top strand (asterisk) were generated by PCR using pGG49 (bps6197/12/23) or its derivatives as templates (see diagrams; designations are indicated at right). Radiolabeled primer 6000F and unlabeled primer 6195 or 6197 (arrows) were used to amplify DNA fragments used for markers in panel (B). For PCR, Pfu polymerase was used instead of Taq. (B) The DNA fragments described in (A) were subjected to in vitro cleavage by WT cMR1/cMR2 in the absence or presence of HMGB1 as indicated. Reaction products were fractionated on a 40% formamide sequencing gel in parallel with PCR-generated markers in (A). Expected fragment sizes and compositions are indicated at left and right, respectively
