# Supplementary material for: HMGB1/2 can target DNA for illegitimate cleavage by the RAG1/2 complex
Source: BMC Mol Biol. 2009 Mar 24;10:24. doi: 10.1186/1471-2199-10-24 (PMC2666730; doi:10.1186/1471-2199-10-24)
Supplement: Additional file 2 — RAG-mediated bps6197 cleavage is not affected by the distance or orientation of the 12-RSS partner. (A) Diagrams of PCR-generated substrates containing bsp6197 and a 12-RSS in cis positioned proximally or distally in the same or reverse orientation. (B) Sequencing gel showing reaction products from RAG-mediated cleavage of PCR-generated substrates. [file 1471-2199-10-24-S2.pdf]

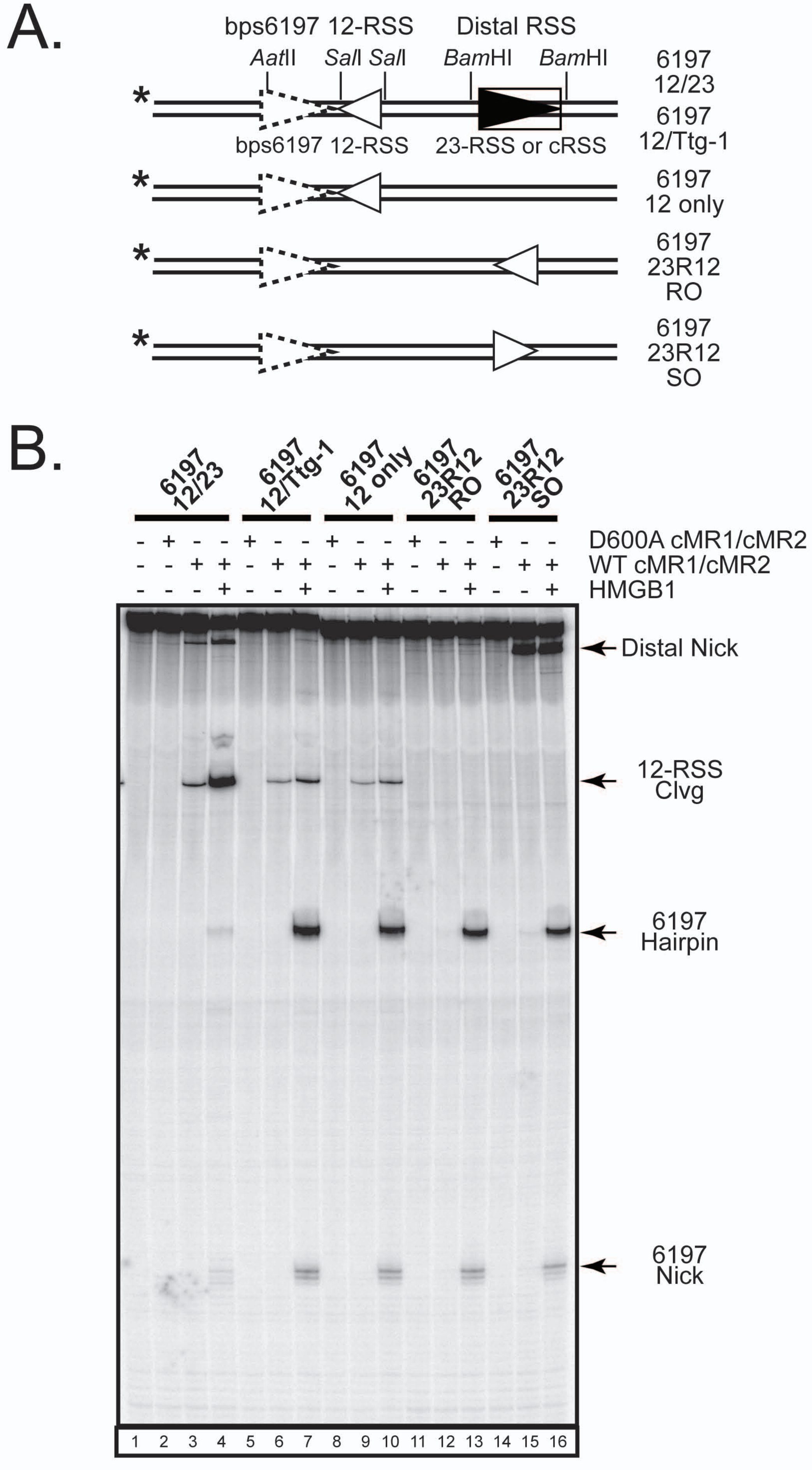

Additional Data File 2. RAG-mediated bps6197 cleavage is not affected by the distance or orientation of the 12-RSS partner. (A) DNA fragments radiolabelled at the 5′ end of the top strand (asterisk) were generated by PCR using pGG49 (bps6197/12/23) or its derivatives as templates (see diagrams; designations are indicated at right). (B) The DNA fragments described in (A) were subjected to in vitro cleavage by WT or D600A cMR1/cMR2 in the absence or presence of HMGB1 as indicated. Reaction products were fractionated on a 40% formamide sequencing gel. Expected fragment compositions are indicated at right.
