# Supplementary material for: HMGB1/2 can target DNA for illegitimate cleavage by the RAG1/2 complex
Source: BMC Mol Biol. 2009 Mar 24;10:24. doi: 10.1186/1471-2199-10-24 (PMC2666730; doi:10.1186/1471-2199-10-24)
Supplement: Additional file 3 — Supplementary Materials and Methods. Construction of variants of pGG49 and pJH299 plasmid V(D)J recombination substrates described in the text. [file 1471-2199-10-24-S3.pdf]

Additional Data File 3: Supplementary Materials and Methods

DNA constructs: Derivatives of pGG49 in which the 12-RSS is removed and the 23-RSS is replaced by a 12-RSS in the same orientation or reverse orientation (6197/23R12SO and 6197/23R12RO, respectively; see Additional Data File 2: RAG-mediated bps6197 cleavage is not affected by the distance or orientation of the 12-RSS partner) were prepared by removing the 23-RSS from the 6197/23 only substrate (see Fig. 2) by BamHI digestion and ligating the plasmid backbone to an oligonucleotide duplex prepared by annealing oligomers 23R12Top (5'-GATCCCACAGTGATACAGCGCTTAACAAAAACCCTCGG-3') to 23R12Bot (5'-GATCCCGAGGGTTTTTGTTAAGCGCTGTATCACTGTGG -3'). After transformation, the clones were screened for the presence of an engineered AfeI restriction site and sequenced to verify the orientation of the 12-RSS.

To generate variants of pGG49 containing mutations in the inverted repeat sequence (6197 mIR, see Fig. 4A), a DNA fragment was amplified from pGG49 by PCR with primers 6000F (5'-TATTGTCTCATGAGCGGATAC-3') and 6103R (5'-TTCTTAGACGTCAGGTGGGTATTTTCGGGGAAATG). The PCR product was cloned using the Topo-TA cloning kit (Invitrogen, Carlsbad, CA) and the sequence verified. A DNA fragment containing the mutated inverted repeat sequence was introduced into pGG49 or its derivative lacking a 23-RSS (6197/12 only) by cassette replacement using BsrBI and AatII. To generate version of pGG49 containing mutations in the bps6197 nonamer motif (see Fig. 4C), a DNA fragment was amplified from pGG49 by PCR using primers mNonF (5'-CCACCTGACGTCTAAGAAACCATTATTATCATGGGTGCTTAATATAAAAATA

GGCGTATCAGC-3') and 6624R (5'-GAACGGTGGTATATGGAGTG-3'). The PCR product was cloned as described above and introduced into pGG49 or its derivative lacking a 23-RSS by cassette replacement using AatII and SpeI.

Derivatives of pJH299 in which the 23-RSS is replaced by the bps6197 sequence in the same or reverse orientation (12/6197SO and 12/6197RO, respectively; see Fig. 7) was generated in two steps. In the first step, the bps6197 sequence was removed from pJH299 by amplifying a DNA fragment from pJH299 by PCR using primers 2495F (5'-GGTTCCTTCATGCATAAAGTG-3') and 4023R (5'-CACCTGACGTCGGCGTATCACGAG-3'). The PCR product was cloned and sequenced as described above, and introduced into pJH299 by cassette replacement using AatII and NsiI. In the second step, the 23-RSS was replaced by the bps6197 sequence by removing the 23-RSS by BamHI digestion, ligating the plasmid backbone to an oligonucleotide duplex prepared by annealing oligomers bps6197Top (5'-GATCCAAAGTGCCACCTGACGTCTAAGAAACCATTATTATCATGACATTAACCTATG-3') and bps6197Bot (5'-GATCCATAGGTTAATGTCATGATAATAATGGTTTCTTAGACGTCAGGTGGCACTTTG-3') After transformation, the clones were screened for the presence of an AatII restriction site and sequenced to verify the orientation of bps6197.
